# Differences Between Readmitted and Non-readmitted Women in an Italian Forensic Unit: A Retrospective Study

**DOI:** 10.3389/fpsyg.2021.708873

**Published:** 2021-10-20

**Authors:** Ilaria Rossetto, Massimo Clerici, Filippo Franconi, Alan R. Felthous, Fulvio Carabellese, Giancarlo Di Vella, Maria Gloria Gandellini, Lia Parente, Felice Carabellese

**Affiliations:** ^1^Poli-REMS Castiglione delle Stiviere, ASST Mantova, Mantova, Italy; ^2^UOC of Psychiatry, Università Milano Bicocca, Milano, Italy; ^3^Forensic Psychiatry Division, Department of Psychiatry and Behavioral Medicine, Saint Louis University School of Medicine, St. Louis, MO, United States; ^4^Department of Medical Sciences, Surgery and Neurosciences, University of Siena, Siena, Italy; ^5^Department of Medical Legal, University of Torino, Turin, Italy; ^6^Formerly Poli-REMS, ASST Mantova, Mantova, Italy; ^7^Section of Criminology and Forensic Psychiatry, Department of Interdisciplinary Medicine, University of Bari Aldo Moro, Bari, Italy

**Keywords:** discharge, female forensic psychiatry inmates, readmitted, non-readmitted, personality disorders, substance use

## Abstract

The main objective of this study was to compare readmitted (RW) and non-readmitted (NRW) female psychiatric patients after being conditionally or unconditionally released from Italian inpatient forensic psychiatry services, in order to identify variables that were significantly linked with readmission. This study included all patients who were discharged from the female Residences for the Execution of the Security Measure (REMS) of Castiglione delle Stiviere from January 2008 to June 2015 who were not readmitted until December 31, 2018 (48). In addition, data were collected on female patients who were discharged from the same REMS before 2008 and readmitted from January 2008 to December 2018 (42). A key finding of our study was that the readmission into a female REMS was positively associated with the presence of substance use disorders (SUD) and a primary diagnosis on Axis II. To a lesser extent, younger age, being unconditionally discharged when first released, having had a shorter length of inpatient stay and having committed a crime against property for the first REMS admission was also variables that were apparently linked with readmission. The present research continues the previous research on gender-specific mentally ill offenders. Hence, the decision to proceed separately with a sample of men only and one of women only. For all these reasons, young female patients with personality disorder and SUD perhaps should remain longer in REMS and be released with conditions. In most European countries, the length of stay depends on the clinical condition and risk assessment, with some exception*s* where the courts set a maximum length of stay at the outset, as in Italy. All the factors listed above influence the risk assessment. Finally, from integrating these findings into the increasing international literature on conditional release and considering the recent changes in the Italian forensic treatment model, we recommend continuing research on individual risk and protective factors as well as risk assessment instruments on conditionally and unconditionally released inpatients with genders studied separately.

## Introduction

### The Italian Forensic Mental Health System

In the past 6years, research on conditional release has continued to increase (Vitacco et al., 2014; Green et al., 2016), yet conditionally released female insanity acquittees continue to be understudied. Outcomes in conditional release are of special interest in Italy where the country has replaced its large forensic psychiatric hospitals *Ospedale Psichiatrico Giudiziario* (OPG) with small local secure treatment facilities *Residenze per l’Esecuzione delle Misure di Sicurezza* (REMS). After the closure in Italy of the psychiatric hospitals (*Ospedale Psichiatrico*=OP) occurred more than 40years ago, this new model is in harmony with the care model of general psychiatry. The REMS is essentially residential community, which is integrated within the larger community model of general psychiatry. The Law 9/2012 ordered in fact the closing of OPGs and the change to a model of care based on regional residential units in the community using only clinical staff incorporated into the public mental health services (Carabellese and Felthous, 2016; Scarpa et al., 2017). Because Italy chose an approach to the care of mentally ill patients that is different from the other European countries (closure of psychiatric hospitals, short hospitalizations, on average 15days, outpatient rehabilitation and resocialization activities, economic and working support for the patient, social and economic support for families, and resistance against stigma), during these years, Italian psychiatrists acquired specialized skills that characterize the operating practices to which they apply as: Some of the strengths of the current Italian mental healthcare model include the widespread public outpatient psychiatric services throughout the country and direct access to the public general psychiatric services for patients, interventions by the family, and social environment and attention to other protective prognostic factors (the quality and variety of intra- and extra-family relationships, working and living independence, the regularity and frequency of contacts with the services that support the patient, and the constancy of care).

These strengths are useful in the treatment of mentally ill individuals in Italy and equally so, it was thought, of socially dangerous mentally ill individuals who are treated in the community. It was hoped that attention to such protective factors would contribute to a reduction in the risk of future criminal behavior in mentally ill offenders and promote their social reintegration into their home environments. There is evidence in the literature (Catalano et al., 1998; Furstenberg et al., 1999) that suggests that some psycho-social factors exert a protective effect. In addition, some patients can be efficaciously influenced through intervention, while in other cases, intervention is more complex and less effective. An assessment of this kind, however, implies not only in-depth knowledge of the patient’s profile, but also the identification of all those variables (family, social, and context-related) that can influence the patient’s behavioral choices (Carabellese et al., 2015). But, of course, there could be some weaknesses, foremost the fact that after the OP closures clinical psychiatrists did not manage violence risk assessment. Moreover, because this new forensic treatment system had not been tried before, its actual benefits and liabilities remained still untested.

The REMSs are residences with low to medium security compared with forensic facilities in other European countries. The REMSs are small residences where patients live assisted by health personnel 24h a day. Inside the REMS, patients participate in therapeutic, treatment, and rehabilitation activities: They regularly are treated with pharmacotherapy under the supervision of health personnel, individual psychotherapy activities, and/or of groups, they take care of their personal hygiene. Those with substance use problems naturally are provided with more specific treatment plans, and they participate in psychoeducational activities in which their family members are also involved. Extra-clinical activities are many and include the acquisition of social skills, from the simplest (buying daily consumer goods) to the most complex ones (knowing how to use public transportation, participating in cultural, educational, and job training activities). They also include participation in physical activities and, as long as there are no specific prohibitions, they are allowed to spend time outside of the facility with their family. The importance of comprehensiveness of therapeutic interventions in forensic psychiatry is highlighted in the EPA guidelines (Völlm et al., 2018). By law, internment in REMS is a custodial security measure which is “extreme and exceptional” and in any case, Law 81 of 2014 limits the maximum duration of internment in REMS to the maximum time of imprisonment had the offender been found guilty of the crime and sentenced.

In December 2018 in 30 out of 31 REMS existing in all 20 regions of Italy (Region is the first administrative body of the State), there were 604 committed inpatients, 80 of whom were women (Catanesi et al., 2019) less than half of total inpatients in the six OPGs before their closures. The number of REMS per region depends on the population of the individual region; thus, some regions only have one REMS and others have several.

Admission into a REMS can only take place for offenders acquitted because of a finding of *infermità*, i.e., insanity, a mental disorder, and compulsory referral by the Judge of Preliminary Investigations (*Giudice delle Indagini Preliminari*) or the Surveillance Court (*Magistrato di Sorveglianza*) or, but only as a provisional security measure, by the prosecutor. The security measure is usually adopted after the evaluation by at least one forensic psychiatrist, sometimes two or three, chosen at the discretion of the judge.

When the patient restricted to REMS is considered by the REMS psychiatrists to have been rehabilitated, or no longer at risk of criminal recidivism, the psychiatrists propose to the judge that patient be discharged from the REMS. At that point, the custodial security measure can be revoked completely, and the patient becomes free again. If, on the other hand, health professionals believe that the risk of the patient’s criminal recidivism still persists, although reduced, the patient can be discharged from REMS but subjected to a non-custodial security measure, the *libertà vigilata*, “Conditional Release.”

Upon discharge from a REMS, many patients are subject to conditions under the law of the Penal Code (*Codice Penale,* art. 228). The judge, usually of the Surveillance Court, is the only authority that can also apply this other security measure. On conditional release, patients must leave the REMS, but if they violate the restrictions decided by the Court they may be readmitted. Restrictions with which the patient must comply and which are written into the conditional release order are medication compliance, collaborating with supervision from mental health services, continuing to pursue the planned resocialization and rehabilitation activities, living in a specific location, not going to other places, respecting certain rules, and certain prohibitions (such as not taking illicit substances, for example, not staying away from one’s residence beyond a certain time in the evening, not associating with individuals who have committed crimes, and not leaving the city in which they live).

In the Lombardy Region, there is the REMS of Castiglione delle Stiviere, where in the past, there was one of the six Italian OPGs. REMS in Castiglione delle Stiviere actually consists of eight REMSs, with a total of 160 beds, one of which admits only women. REMS of Castiglione delle Stiviere serves all of Lombardy (more than 10 million inhabitants). As far as we are aware, in Italy, there are no other REMS for only women like that of Castiglione delle Stiviere. However, Castiglione has a long tradition in this regard, having managed since 1975 the only section in OPG for all socially dangerous women that existed in Italy until the end of 2014. This section remained open until March 2015, when all OPGs were definitively closed and it continued to be the only one in Italy to have socially dangerous women until then. By June 30, 2010, the Ministry of Justice had 95 women in the female section of the OPG of Castigione delle Stiviere out of a total of 1552 inpatients: 1457 men and 95 women. During the closing of the OPGs, inpatients were progressively transferred to the REMSs. The numbers of patients inside the six OPGs fell progressively until their final closures; at the end of 2014, there were 672 inpatients in the six OPGs.

Despite the fact that women occupied 10–12% of the regional secure beds, there remains a shortage of clinical and legal data on females in REMS. At the same time, it is important to underscore how women confined in the OPG and currently in the REMS are always very few compared to men and the specific aspects related to gender only in recent years have been investigated by our group (Carabellese et al., 2018, 2019a,b, 2020).

Data in the international literature pertain mainly to male patients and in a few investigations conducted on new forensic facilities in Italy (De Girolamo et al., 2016; Scocco et al., 2019; Carpiniello et al., 2020).

In line with our previous surveys, in order to better investigate any gender factors related to REMS readmissions, in such a unique and recently established forensic treatment model as is the Italian model, we also wanted to investigate the sample of women separately.

As for significant outcomes in forensic services, the average length of stay of these patients in forensic hospitals is 3years, but the death rate, the readmission rate, and the reoffending rate show substantially high diversity worldwide (Fazel et al., 2016). Comparisons between international studies are problematic because of variations in many essential specifics, including settings, laws, descriptions of populations, outcome measures, and follow-up periods (Lund et al., 2012; Di Lorito et al., 2017; Mandarelli et al., 2019; Slamanig et al., 2021). Notwithstanding this, there is some evidence that patients discharged from forensic psychiatric services have lower rates of criminal recidivism than comparative groups (Hayes et al., 2014; Charette et al., 2015; Norko et al., 2016). This is to be balanced against the realization that for some crimes, as recently published data revealed, forensic inpatients remain in isolation longer than mentally healthy perpetrators of similar offenses (Gosek et al., 2021).

In Italy, only two studies were published that focused on this topic (Russo, 1994; Fioritti et al., 2001) and both before the recent changes briefly described. Moreover, only the first one analyzed patients discharged from the High Security Hospital (OPG) of Barcellona Pozzo di Gotto in Sicily and then readmitted to the same hospital after committing a new crime. The other was a preliminary study on the process of closing the Italian OPGs.

In light of the context of treatment of the forensic and general psychiatric treatment model briefly described above, the main objective of this present study was to identify variables in female patients that are significantly linked with readmission to REMS. The initial hypothesis was that the use of substances, a personality disorder, the length of stay in REMS, and discharge without conditional release are risk factors for REMS readmission. Another goal we proposed was to ascertain any gender specificities in discharged patients that we believed could be better appreciated by studying women from men given their enormous numerical disparity. In order to confirm this, we compared female psychiatric patients who were readmitted and those who were not readmitted after having been conditionally or unconditionally released from the REMS.

### International Studies

#### Characteristics of Women in Secure Inpatient Facilities

Although not clearly mental illness acquittees, admission characteristics of women in secure inpatient facilities in the United Kingdom were compared with those of male inpatients by Archer and colleagues (Archer et al., 2016). Women admitted to these facilities had fewer previous convictions, more previous psychiatric hospitalizations, and were more likely to have been transferred from a hospital than a prison. Women were more likely to have been charged or convicted of arson. Whereas males were more likely to have been diagnosed with schizophrenia and co-occurring substance misuse, the more commonly diagnosed mental disorders among female inpatients were major depressive disorder, borderline, and other personality disorders. In comparison with male inpatients, women were more likely to have had a history or physical and sexual abuse (Archer et al., 2016).

#### Adverse Outcomes Following Discharge From Secure Psychiatric Hospitals

Fazel and colleagues recently reported a follow-up review and meta-analysis of patients discharged from secure psychiatric hospitals in which they registered adverse outcomes. This study systematically reviewed 35 studies from 10 countries with a total of 12,056 patients, 53% of whom had been violent offenders. They found the crude death rate for all causes of mortality to be 1,538 per 100,000 person years with a crude rate of suicide of 325 per 100,000 persons years (the types of hospital readmission were not further specified), a readmission rate of 7,208 per 100,000 person years, and crude reoffending rate of 4,484 per 100,000 person years (Fazel et al., 2016). This study identified significant post-discharge risks, of which death and suicide are especially concerning in this population but did not distinguish whether discharges occurred within the context of stepdown and conditional release programs.

Maden and colleagues compared men and women’s reoffending rates following discharge from medium-secure units (Maden et al., 2006). Although not designated as such, one could infer that these were mostly medium-secure hospital units, not ordinary prison facilities. The legal statuses of these offenders were not specified. Included in the study were 843 men (88%) and 116 women (12%) from 34units. They were followed for 12months following discharge or transfer, except that reconviction data were extended for 2years. This was not a study of success or failure following discharge but provided useful information about potential gender differences in male and female offenders who are treated following their release.

In this study, gender differences were identified upon admission to the units. Women self-reported prior physical and sexual abuse and self-harm more frequently than men. They were also more likely to have been admitted with a personality disorder and to have been treated psychiatrically than men. Their index offenses were less likely to have been property or sexual crimes, and they were less likely to have been convicted previously two or more times and to have served prior prison sentences in comparison with the male offenders.

Women were less likely to be re-convicted following discharge than men. Independent predictors of reconviction were age, self-harm, history of drug problems, and prior convictions. These findings were consistent with the literature showing that history of self-harm is associated with a lower risk of reconviction, whereas sexual abuse is associated with a higher risk as is a history of alcohol and drug abuse (Maden et al., 2004). Also consistent with prior studies is the predictive value of previous convictions; female offenders less often have this history (Maden et al., 2006).

#### Female Insanity Acquittees

An early attempt to examine female insanity acquittees apart from males was the series of studies reported by Rogers and colleagues on Oregon State’s Psychiatry Security Review Board (Rogers et al., 1986). The PSRB provided a comprehensive and continuous system for monitoring and managing insanity acquittees who were discharged from the hospital on conditional release. Women were conditionally released at a significantly higher rate. A larger percentage of women had been charged with misdemeanors, but for those charged with a felony the offense was homicide or attempted homicide. As was observed in our prior studies (Carabellese et al., 2019a), female insanity acquittees were underrepresented among those whose crimes that involved strangers. During the study period, a higher percentage of female insanity acquittees was discharged (Rogers et al., 1986).

#### Female Insanity Acquittees Placed on Conditional Release

To our knowledge, the first and most comprehensive follow-up study of female insanity acquittees placed on conditional release was that by Vitacco and colleagues. The investigators studied 76 female insanity acquittees who were conditionally released in the State of Wisconsin over a 7years period. Individual subjects were followed for 3–7years. Forty-one of the females (53.9%) had been found NGRI for a violent offense, 6 (7.9%) for murder. A formal psychological risk assessment instrument was not used for determining level of supervision. Common diagnoses were schizophrenia (44.7%), bipolar disorder (23.7%), and depression (11.8%), but personality disorders (39.5%) and comorbid substance abuse (34.2%) were also found.

A significant finding, using logistic regression and conditional release as the dependent variable, was that the conditional release of females who required short-term hospitalization was more likely to be revoked based on violating the terms of the conditional release or non-violent criminal activity (*p*=0.002). Based on this finding, the authors recommend a strategy of increasing mandated mental health services at the first sign of exacerbation and before hospital care becomes necessary. Although most of these females had been found NGRI for a violent offense, none of their conditional release revocations were based upon a violent offense. Most (68.4%) successfully maintained their conditional release. Of those who had their conditional release revoked, six were released again and five of these had their conditional release revoked again (Vitacco et al., 2011). Since this report, other studies confirmed that a history of conditional release revocation is a predictor of future CR revocation.

Age and diagnosis alone were not predictive of CR revocation; however, a model that included these factors (age, mood disorder, number of charges, short-term hospitalization, and supervision level) was weakly associated and accounted for 15.8% of the variance [wald=9.82, *X*^2^(5)=13.08, *p*=0.02; Vitacco et al., 2011].

#### Studies of Insanity Acquittees Who Were Conditional Released

Comparison with other outcome studies of insanity acquittees placed on conditional release is difficult because of differences in dependent and independent variables, lengths of study periods, and variability in the detailed descriptions of the studied subjects and the stepdown, and conditional release procedures. Moreover, the nature and extent of the inpatient and outpatient treatment are typically not described. Female insanity acquittees who are conditionally or unconditionally released are vastly understudied.

In a retrospective study, Green and colleagues examined 142 insanity acquittees who had been transferred from a forensic hospital in New York state within 10years. Of the 40 who were recommitted, 12.5% were female; of the 102 non-recommitted, 30.4%. Having applied the HCR-20, the investigators found that only the Historical scale was associated with recommitment; however, only a few individual items within this scale were risk factors for recommitment. Those factors which were informative in predicting recommitment over 10 and 3years periods were less serious major mental illness, relationship problems, problems with substance use, negative attitude, and prior supervision failure.

Lund and colleagues conducted a 2years study on mentally disordered male offenders in Sweden, 152 of whom were treated in a forensic psychiatric facility (FPT), 116 in prison, and 50 with non-custodial sanctions. Only those who were in the FPT and placed on conditional release showed significantly lower rates of criminal recidivism. Similar to the present study is findings of female insanity acquittees, recidivism was significantly more common in offenders with either a substance abuse or personality disorder than with psychotic or other mental disorders alone. Also predictive of recidivism was age at index crime and number of prior criminal offenses. The authors found that the level of supervision was more predictive of post-release success than individual factors (Lund et al., 2012).

Manguno-Mire and colleagues followed 193 individuals (151 males and 42 females) who were placed on conditional release having been found incompetent to stand trial. Their definition of an “incident” included psychosis relapse, substance abuse relapse, treatment non-adherence, or becoming absent from follow-up, rule, or curfew violation and arrest (Bertman-Pate et al., 2004). Seventy percent of these individuals maintained their conditional release. Success was predicted by the individual’s financial resources, not having a personality disorder and having few incidents. Striking was the difference in number of days until first incident between those placed on conditional release from jail versus from the forensic security hospital (67 vs. 575days; Manguno-Mire et al., 2014).

Although in contrast to the earlier study by the New Orleans Forensic Aftercare Clinic, which found no difference in time to first incident, between security hospital and jail discharged individuals (Bertman-Pate et al., 2004), this recent finding suggests that where individuals were last in inpatient treatment can affect the success of conditional release.

For 356 insanity acquittees placed on conditional release upon discharge from forensic hospitals in the state of Maryland, Marshall and colleagues compared those readmitted to the forensic hospital voluntarily (*n*=83) with those readmitted involuntarily (*n*=112). Females constituted 22% of the subjects. Insanity acquittees with fewer arrests (*p*=0.001) and fewer instances of treatment non-compliance (*p*=0.04) were more likely to have been readmitted voluntarily; thus, arrests and treatment non-compliance predicted involuntary readmission. A third group of insanity acquittees was not readmitted to a forensic hospital (*n*=161). When compared with all who had been readmitted, either voluntarily or involuntarily, this group appears to have adjusted better to community living as suggested by significantly fewer community psychiatric admissions (*p*=0.035) and longer duration in the community before any psychiatric readmissions (*p*<0.001) (Marshall et al., 2014).

A study of large sample size was the Canadian National Trajectory project which examined 1,800 men and women in three provinces over 3years. Rates of recidivism varied between provinces (10, 9, and 22%). In all three provinces, those who were released and then followed under the supervision of review boards and those whose index offenses were less severe were less likely to reoffend (Charette et al., 2015).

Monson and colleagues studied the outcome of insanity acquittees discharged from the hospital from January 1, 1985 to December 31, 1998. Of the 201 patients discharged during this period, sufficient records existed on 125 for inclusion. Three factors were shown to be sufficiently predictive of revocation of conditional release: minority status, diagnosis of substance abuse, and prior criminal history (Monson et al., 2001).

The state of Missouri uses a stepdown, conditional release program that has been well described by Reynolds (2016). This system does not use a structured risk assessment instrument to inform conditional release decisions. Of the 110 forensic outpatients on conditional release who were supervised by the Northwest Missouri Psychiatric Rehabilitation Center over a 3years period, only 7% required rehospitalization. Most of these rehospitalizations were voluntary and did not require revocation of their conditional release. Only one person was convicted of a criminal offense (stealing) and elopement was also rare as well as brief (Reynolds, 2016).

Unique for its exceptionally long period of retrospective follow-up, the study by Norko and colleagues included 365 insanity acquittees in the state of Connecticut who had been supervised by the Psychiatry Security Review Board during a period of over 30years. Of the 177 individuals placed on conditional release, the study registered revocation of CR by the PSRB, arrests while on CR, and arrests after discharge from supervision by the PSRB. Of those individuals discharged from CR (215), 16 percent were rearrested. Community supervision on CR and duration of commitment to the PSRB significantly reduced the risk of rearrest among those who were eventually released from PSRB supervision (Norko et al., 2016).

In a recent study of 101 conditionally discharged patients in England, Jewell and colleagues applied Cox regression survival analyses to identify factors associated with recall from conditional release. Of patients discharged between 2007 and 2013 and followed over an average of 811days, 45 (44.5%) were recalled to the hospital. Factors associated with a shorter time until recalled were younger age, non-white ethnicity, history of substance abuse, early childhood maladjustment, depot medication, and having been known to mental health services. Remarkably, treatment with clozapine reduced the risk of recall (Jewell et al., 2018).

#### Substance Use Disorder and Conditional Release

Although not all studies of conditionally released patients included substance use as a variable, the afore mentioned study by Green and colleagues found problems with substance use to be an informative factor from the HCR-20 with regard to recommitment (Green et al., 2014). Lund’s study in Sweden showed substance use disorder (SUD) was significantly more common in mentally disordered offenders released from a FPT than other mental disorders alone including psychotic disorders (Lund et al., 2012). In the England study by Jewell et al., history of substance abuse was significantly associated with recall from conditional release (Jewell et al., 2018). The national UK study by Maden et al. (2006) found reoffending after discharge from medium security units to be associated with a history of drug problems. Of studies that included substance abuse, this factor has consistently been associated with failure on conditional release (Cohen et al., 1988; Callahan and Silver, 1998; Monson et al., 2001; Vitacco et al., 2008, 2014; Green et al., 2014). Apart from conditional release of insanity acquittees, substance abuse is one of the strongest predictors of general criminal recidivism among mentally disordered offenders (Bonta et al., 1998) and, together with a history of violence, of future violent behavior (Swanson, 1994; Swanson et al., 2000; Douglas and Skeem, 2005; Conroy and Murrie, 2007, Hanson, 2009, see generally Tabernik and Vitacco, 2016).

Tabernik and Vitacco postulated several explanations for the association between substance use and failure at conditional release: The association of substance use with forms of criminal conduct, the potential for substance use to exacerbate a mental disorder, and substance use *per se* can be reason enough to revoke conditional release (Tabernik and Vitacco, 2016). We should add a possible association with medication non-compliance and the potential for substance use alone inducing a mental state that predisposes the individual to criminal conduct or rule violation (e.g., intoxication or substance induced psychotic disorder).

#### Formal Risk Assessment

Risk assessment informs decisions on conditional release. Four approaches to assessing aggressive and violent behavior which have relevance to risk assessment are as: clinical, actuarial, behavioral, and phenomenological (Felthous, 2010, 2013). Each has its own strengths, specific applications, and limitations. Today structured professional judgment (Murrie and Agee, 2018) is commonly recommended. This approach incorporates but does not completely rely on a risk assessment instrument. Structured risk assessments include the Classification of Violence Risk (Monahan et al., 2006), the Violence Risk Appraisal Guide (Quinsey et al., 2006), and the HCR-20. The Psychopathy Checklist-Revised (PCL-R, Hare, 1991) is shown to predict criminal recidivism (Hare, 2007, 2020 in press).

Most outcome studies of conditional release do not assess the utility and predictive validity of risk assessment instruments. Of those that have included a risk assessment instrument, the HCR-20 has received studied attention (Douglas, 2014). Performance results thus far have been mixed. As already noted, Green et al. found significant associations with revocation of conditional release with the Historical scale specific items on the Historical scale but not on the Clinical or the Risk scale (Green et al., 2014). In the study by Vitacco et al. (2014), only factors under Risk predicted failure on conditional release, previous failure on conditional release, and poor hospital treatment adherence. Demographic and criminologic factors were not significantly associated with CR failure. Three items were significantly associated with earlier revocation: previous failure on conditional release, number of prior charges for violent offenses, and total number of charges (Vitacco et al., 2014). In a subsequent study of 116 forensic inpatients who were assessed with the HCR-20 prior to conditional release from state forensic facilities, of which 39 were released and returned, 19 were released but not readmitted, and 58 were not released during the seven year study period. In this study, higher scores on the Risk management scale predicted either non-release, or if released, readmission (Vitacco et al., 2016).

Vitacco et al. (2016) point out the following factors as having been predictive of CR failure without administration of a formal risk assessment: substance use, personality disorder, treatment non-compliance, deficient financial support, and need for increased mental health services in the community (Vitacco et al., 2016). More recently, new approaches were described that can be used to facilitate the process of risk assessment, as, for example, the web-based tools like FoVOx (Cornish et al., 2019) or the telepsychiatry (Kennedy et al., 2021).

In the Italian forensic treatment model, little attention has been given to the use of validated assessment tools in general and standard risk assessment instruments have not yet been translated and validated for the Italian population. For example, consider that the HCR 20 V3 was not published in Italian until 2019 (Caretti et al., 2019). It is therefore evident that the judgments on the discharge of patients from REMSs are above all clinical and based on the experience acquired in the treatment of patients with mental disorders who have not committed crimes.

## Materials and Methods

### Sample and Study Setting

The study sample consisted of all patients who were discharged from the female OPG section and, after its closure, the female REMS of Castiglione delle Stiviere from January 2008 to June 2015 and who were not readmitted before December 31, 2018, allowing a minimum of 42months follow-up, with a range from 3½ to 10years. In addition, data were collected on female patients who were discharged from the same REMS before 2008 and readmitted from January 2008 to December 2018. We examined a database of electronic clinical records of all the patients. The data were anonymized. Demographic, clinical, and legal data were routinely collected upon admission and during inpatient care. Individuals who died during their stay in the REMS were excluded.

### Ethics

This research was conducted in compliance with the rules established by the Ethical Committee for the facility, which approved the study in advance.

### Variables

We compared non-readmitted women (NRW) to readmitted women (RW) for each of these variables: Primary diagnosis at first discharge: Axis I vs. Axis II; SUD; crime against the person vs. property crime; conditional release (CR) or unconditional release (NCR); median length of stay; and mean age at first discharge.

### Data Sources

At the time of their first discharge, all patients were given a clinical diagnosis according to the Diagnostic and Statistical Manual of Mental Disorders IV Edition Text Revision (DSM-IV-TR, American Psychiatric Association, 2000). The following psychiatric diagnoses at first discharge were investigated as: schizophrenia spectrum disorders (SSD), mood disorders (MD), SUD, personality disorders (PD), and learning disability (LD). The diagnoses of patients were further divided into Axis I and Axis II according to the DSM-IV TR criteria.

Readmission was defined as re-entry into the REMS after having been discharged (whether or not conditionally). SUD was considered as a primary diagnosis or as a comorbidity because of the increased risk of mortality and because comorbid substance use and personality disorder increases the risk of violent offending (Fazel et al., 2016). In addition, neuropsychiatric factors and above all SUD are the most important risk factors for interpersonal violence in the general population (Fazel et al., 2018).

Reoffending was described as readmission into a REMS for any kind of crime (violent and non-violent) that resulted in a new verdict. Violent reoffending was defined as a crime that was a serious threat to the victim and that resulted in a new verdict. Crimes at first admission were classified as crimes against the person, which included as: Homicide and attempted homicide; aggravated and common assault, sexual offenses, assaulting an officer, kidnapping, threats, and harassment; property crimes, which included as: robbery and arson; and non-violent crimes, such as burglary, traffic and drug offenses, extortion, and revocation of conditional discharge. The difference between crimes against the person and against property was examined because offenders convicted of drug and non-violent offenses have higher rates of reoffending compared to serious offenders (Coid et al., 2009). Unconditional release is the release without judicial restrictions because the patient was deemed to no longer be at risk of reoffending.

### Statistical Analysis

To determine whether there was a statistically significant association between two variables, we first computed the Pearson’s non-parametric chi-squared test between readmitted (/non-readmitted) and nominal variables (primary diagnosis at first discharge, substance use, and crime against – the person/property – conditional release). Then, we computed the Mann-Whitney test between readmitted/non-readmitted and the scale variables (length of stay in months and age in years at first discharge). The chi-squared test was used to verify the null hypothesis that the two variables were independent. We chose a significance level of 0.05; then, we disproved the null hypothesis of independence when the value of p was lower than 0.05. For significance of results, we used the Cramer’s V, which is a measure of dependence between two nominal variables. It uses values from 0, in the case of independence, to 1, in the case of maximum dependence. The Mann-Whitney test verified the null hypothesis that the medians of the chosen scale variables were equal between readmitted and non-readmitted.

### Chi-Square Test and ANOVA

To compare female psychiatric patients who were readmitted with those who were not readmitted after having been conditionally or unconditionally released from the REMS, and thereby the interaction between readmission/non-readmission and release status, a 2×2 factorial design defined as “Condition” was created that was composed of four groups: Group 1: readmitted, conditionally released, Group 2: readmitted, unconditionally released, Group 3: non-readmitted, conditionally released, and Group 4: non-readmitted, unconditionally released.

First, the associations between “Condition” and the three qualitative variables “Primary Diagnosis at first discharge,” “Substance Use: yes/no,” and “Crime against the person vs. Crime against property” were evaluated. Because of these qualitative variables, it was not possible to carry out a two-way ANOVA, therefore, a chi-square test was performed between the variable of interest and the four categories resulting from the intersection between Conditional Discharge and Readmission. The crosstabs were then constructed to perform the chi-square test and evaluate the Cramer V index if the chi-square test was statistically significant.

It was possible to carry out a two-way ANOVA only for the quantitative variables “length of stay” and “age at first discharge” with the two factors “Conditional Release: yes/no” and “Readmission: yes/no.” We then evaluated whether there was an effect on each quantitative variable due to the main effects of the two factors and if there was an interaction between the two factors.

#### Logistic Regression

A binary logistic regression was carried out which had the Readmission variable (Yes=1, No=0) as its dependent variable and the remaining variables, i.e., Primary Diagnosis at first discharge, Substance Use, Crime against, Length of stay (months), Age (years) at first discharge, and Conditional Release (as independent variables). The qualitative variables were introduced in the form of dummy variables. The goal of logistic regression was to determine whether and which variables have a statistically significant effect on the probability of being Readmission Yes compared to Readmission No. The dummy variables were parameterized and the null model contains only the constant between the independent variables. The Nagelkerke’s Pseudo R-squared index equal to 0.531 could explain over 50% of the overall variability of the phenomenon.

## Results

Between 2008 and 2018, three female patients died during their residence in the OPG female section or after its closure, in the REMS, two of whom committed suicide. The number of women discharged during a period of time of 7years and 6months and not readmitted after an average follow-up time of 78.9months was 48, while that of readmitted women during 11years was 42 after an average follow-up time of 44months. In this sample, compared to studies in other western countries (Tully et al., 2019), there was a low representation of ethnic minorities (11.9% non-white ethnicity in NRW and 10.4% in RW). Among women who were readmitted for crimes against the person were three patients readmitted for attempted homicide, two for assault, one for sexual offen*s*es, and 10 for threats and harassment. As for property and non-violent crimes, 19 were readmitted for revocation of conditional discharge, five for robbery, one for burglary, and one for traffic and drug offen*s*es. The clinical and criminal characteristics of NRW and RW (90 patients in total) are shown in Table 1.

**Table 1:** Clinical and legal characteristics of discharged patients.

		Non-readmitted women n %	Readmitted women n%
Primary diagnosis at first discharge	Schizophrenia spectrum disorders *	28 (59%)	15 (35%)
	Mood Disorders *	3 (6%)	0 (0%)
	Substance use disorders *	1 (2%)	1 (3%)
	Personality disorders **	13 (27%)	24 (57%)
	Learning disability **	3 (6%)	2 (5%)
	Total	48 (100%)	42 (100%)
Substance use disorder(s)	Yes	3(6%)	17 (40%)
	No	45 (94%)	25 (60%)
	Total	48 (100%)	42 (100%)
Type of index offense at first discharge	Homicide and attempted homicide +	12 (25%)	7 (17%)
	Aggravated and Common Assault +	5 (11%)	7 (17%)
	Sexual offenses +	1 (2%)	0 (0%)
	Assaulting an officer +	1 (2%)	0 (0%)
	Kidnapping +	2 (4%)	1 (2%)
	Threats and harassment +	14 (29%)	6 (14%)
	Robbery ++	3 (6%)	7 (17%)
	Arson ++	0 (0%)	2 (5%)
	Non-violent crime only ++	10 (21%)	12 (28%)
	Total	48 (100%)	42 (100%)
Conditional release	Yes	34 (70.8%)	20 (47.6%)
	No	14 (29.2%)	22 (52.4%)
	Total	48 (100%)	42 (100%)

*Clinical and legal characteristics of 48 women discharged between January 2008 and June 2015 from the female REMS of Castiglione delle Stiviere and who were readmitted before December 31, 2018 and 42 women discharged before 2008 and readmitted between January 2008 and December 2018*. *=Axis I; **=Axis II; +=Crime against person; and ++=Crime against Property.

### Primary Diagnosis at First Discharge

The primary diagnoses of NRW at first discharge were on Axis I in 67% of cases and in 33% on Axis II, while was 38% of cases Axis I and 62% Axis II in RW (see Table 1). Being readmitted or not readmitted was associated on the axis of the primary diagnosis at first discharge. Among the readmitted, those with an Axis I primary diagnosis were readmitted less than the general readmitted frequency (33.4%<46.7%), while those with an Axis II primary diagnosis were readmitted more than the general readmitted frequency (62%>46.7%). Cramer’s V was equal to 0.239, so there was a weakly significant association between the two variables.

Regarding the interaction between Condition and Primary diagnosis at first discharge, Groups 1 and 2 showed higher percentages (respectively 60 and 54.5%) of Axis 2 diagnoses compared to the Groups 3 and 4. To evaluate the significance of this association, a chi-square test it was performed. The chi-square test was, albeit slightly, not statistically significant (*p*=0.150); therefore, it could not be excluded that the difference in percentages of Primary Diagnosis at first discharge in the various subgroups could be due to chance.

Finally, on logistic regression, the effect of the independent variable Primary Diagnosis at first discharge on the dependent variable Readmission was found to be not statistically significant.

### Substance Use Disorders: Presence Vs. Absence

Substance use disorders was present in 6% of cases in NRW and in 40% of RW (see Table 1). Being readmitted or not readmitted depended on whether or not subjects had a SUD. Cramer’s V was equal to 0.432, so there was a moderate dependence between the two variables.

As regards the intersection between Condition (Readmission and Conditional Release) and Substance Use: yes/no, Group 1 (readmitted, conditionally released) had the higher percentage (60%) compared to Groups 2, 3, and 4 (see Table 2). The chi-square test turned out to be statistically significant at 0.001 level (*p*<0.001); therefore, it was possible to conclude that there was a statistically significant association between the Condition and Substance Use: In this case, we observed that Group 1 had a clearly higher percentage of “yes” than the other Groups.

**Table 2:** Crosstab – condition (readmission, conditional release) and substance use.

			Substance use		Total
			No	Yes	
Condition	Conditionally Released + Not Readmitted (Group 3)	Count	33	1	34
		% within Condition	97.1%	2.9%	100.0%
	Unconditionally Released + Not Readmitted (Group 4)	Count	12	2	14
		% within Condition	85.7%	14.3%	100.0%
	Conditionally Released + Readmitted (Group 1)	Count	8	12	20
		% within Condition	40.0%	60.0%	100.0%
	Unconditionally Released + Readmitted (Group 2)	Count	16	6	22
		% within Condition	72.7%	27,0.3%	100.0%
Total		Count	69	21	90
		% within Condition	76.7%	23.3%	100.0%

### Length of First Stay

**Figure 1:**
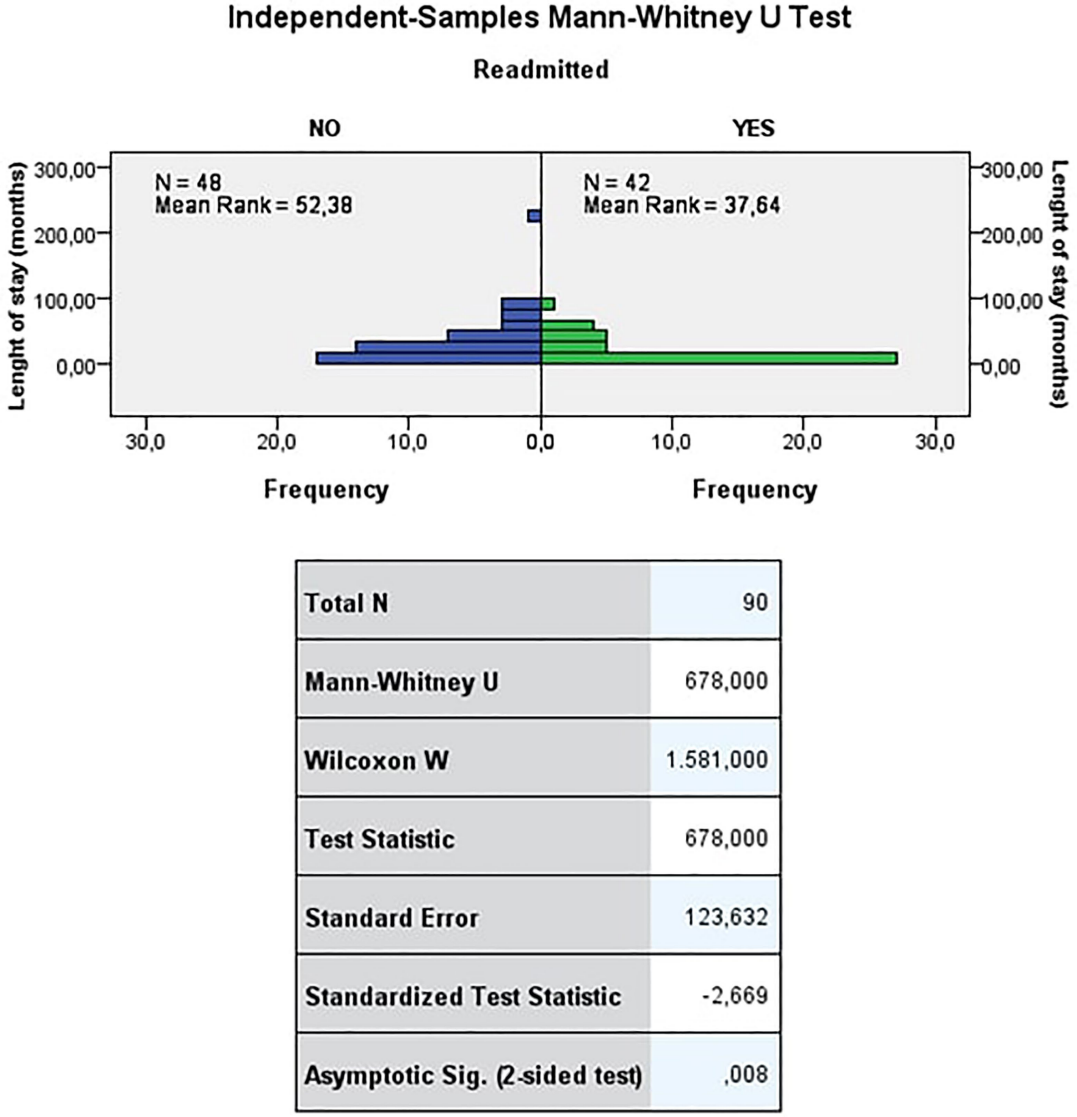
Length of inpatient treatment (months). The Mann-Whitney test refuses the null hypothesis that the distribution of length of stay (months) is the same across the categories of readmitted at a significance level of 0.01 (value of *p* <0.01). Thus, being readmitted or not readmitted depends on the length of stay. Those who were not readmitted had, on average, significantly longer lengths of stay than those who were readmitted.

The median length of inpatient treatment was 26.3months for NRW and 9.6months for RW. Being readmitted or not readmitted was associated with the length of inpatient treatment. Those who were not readmitted had been treated in the REMS significantly longer than those who were readmitted (see Figure 1).

As regards the quantitative variable Length of first stay in the ANOVA analysis, the effects which were statistically significant can be observed in the table Tests of Between-Subjects Effects (Table 3). The main effect of Readmission turned out to be statistically significant at level 0.01 (*p*=0.007) while the interaction effect Condition (Readmission and Conditional Release) turned out to be statistically significant at level 0.10 (*p*=0.056). The main effect of Conditional Release was instead not significant.

**Table 3:** Tests of between-subjects effects **–** dependent variable: length of stay (months).

Source	Type III sum of squares	df	Mean Square	F	Sig.	Partial eta squared
Corrected model	9213,896^a^	3	3071.299	3.225	0.026	0.101
Intercept	67783,593	1	67783.593	71.172	0.000	0.453
Conditional release	472,127	1	472.127	0.496	0.483	0.006
Readmission	7236,313	1	7236,313	7.598	0.007	0.081
Conditional release * readmission	3579,128	1	3579.128	3.758	0.056	0.042
Error	81905,851	86	952.394			
Total	158512,900	90				
Corrected total	91119,746	89				

a *R-Squared = 0.101 (Adjusted R-Squared = 0.070).*

**Figure 2:**
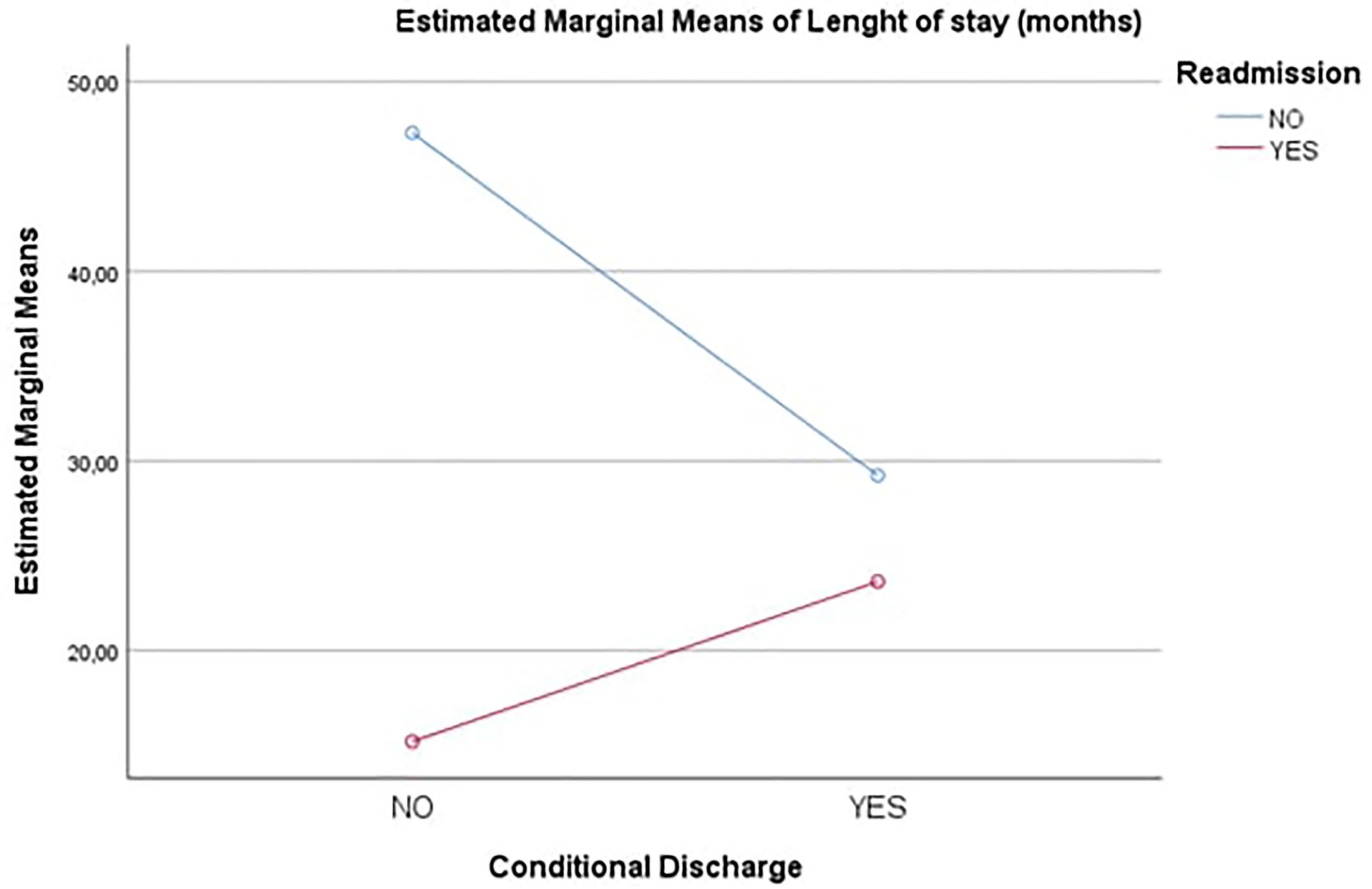
Estimated marginal means – Length of stay (months). Conditional Discharge=Conditional Release.

The differences in the main effects were observed by evaluating the Estimated Marginal Means (see Figure 2). There were two completely opposite trends: as regards the Readmission group “no,” there was a significantly higher value of Length of first Stay when Conditional Release was “no” compared to when Conditional Release was “yes.” On the contrary as regards the Readmission group “yes,” there was a lower value of Length of first stay when Conditional Release was “no” compared to when Conditional Release was “yes.” The fact that the two lines were non-parallel was confirmation of the significance of the interaction effect between Readmission and Conditional Release.

### Mean Age at First Discharge

**Figure 3:**
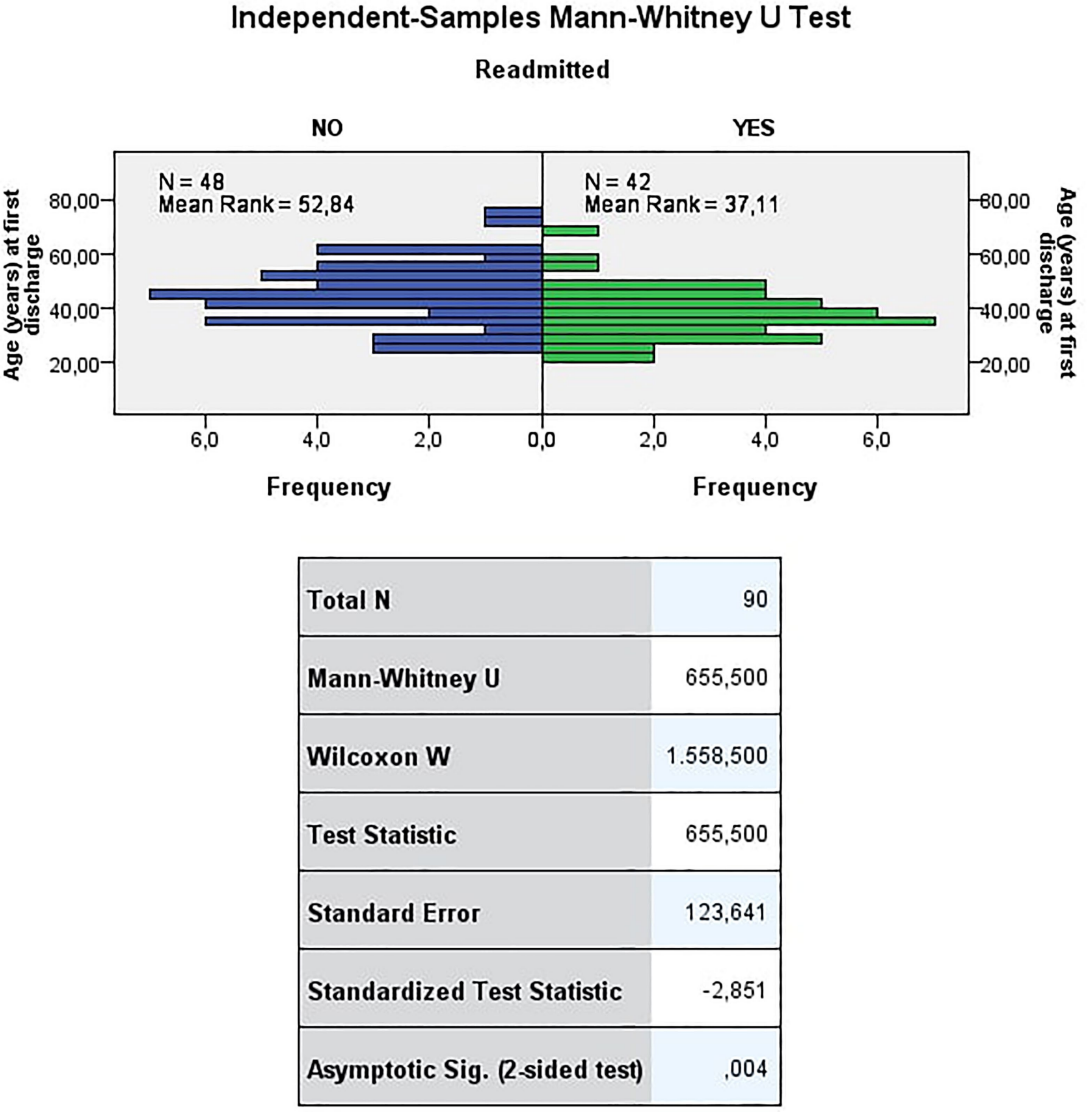
Age (years) at first discharge. The test refuses the null hypothesis that the distribution of the age (years) at first discharge is the same across the categories of readmitted at a significance level of 0.01 (value of p < 0.01). Thus, being readmitted or not readmitted depends on the age at first discharge. Those who were not readmitted were, on average, significantly older than those who were readmitted.

The mean age at first discharge was 45years for the NRW and 38.2years for the readmitted women. Being readmitted or not readmitted depended on the age at first discharge. Those who were not readmitted were, on average, significantly older than those who were readmitted (see Figure 3).

In the ANOVA analysis, the effects that were statistically significant were evaluated by looking at the Tests of Between-Subjects Effects (see Table 4). It can be noted how only the main effect of Readmission proved to be statistically significant at the level of 0.01 (*p* =0.008).

**Table 4:** Tests of between-subjects effects – dependent variable: Age (years) at first discharge.

Source	Type III sum of squares	df	Mean square	F	Sig.	Partial eta squared
Corrected model	1322.788^a^	3	440.929	3773	0.013	0.116
Intercept	138960.803	1	138960.803	1189.124	0.000	0.933
Conditional release	30.922	1	30.922	0.265	0.608	0.003
Readmission	859,894	1	859.894	7.358	0.008	0.079
Conditional release * readmission	238.530	1	238.530	2.041	0.157	0.023
Error	10049.942	86	116.860			
Total	168632.690	90				
Corrected total	11372.730	89				

a *R Squared = 0.116 (Adjusted R Squared = 0.085).*

**Figure 4:**
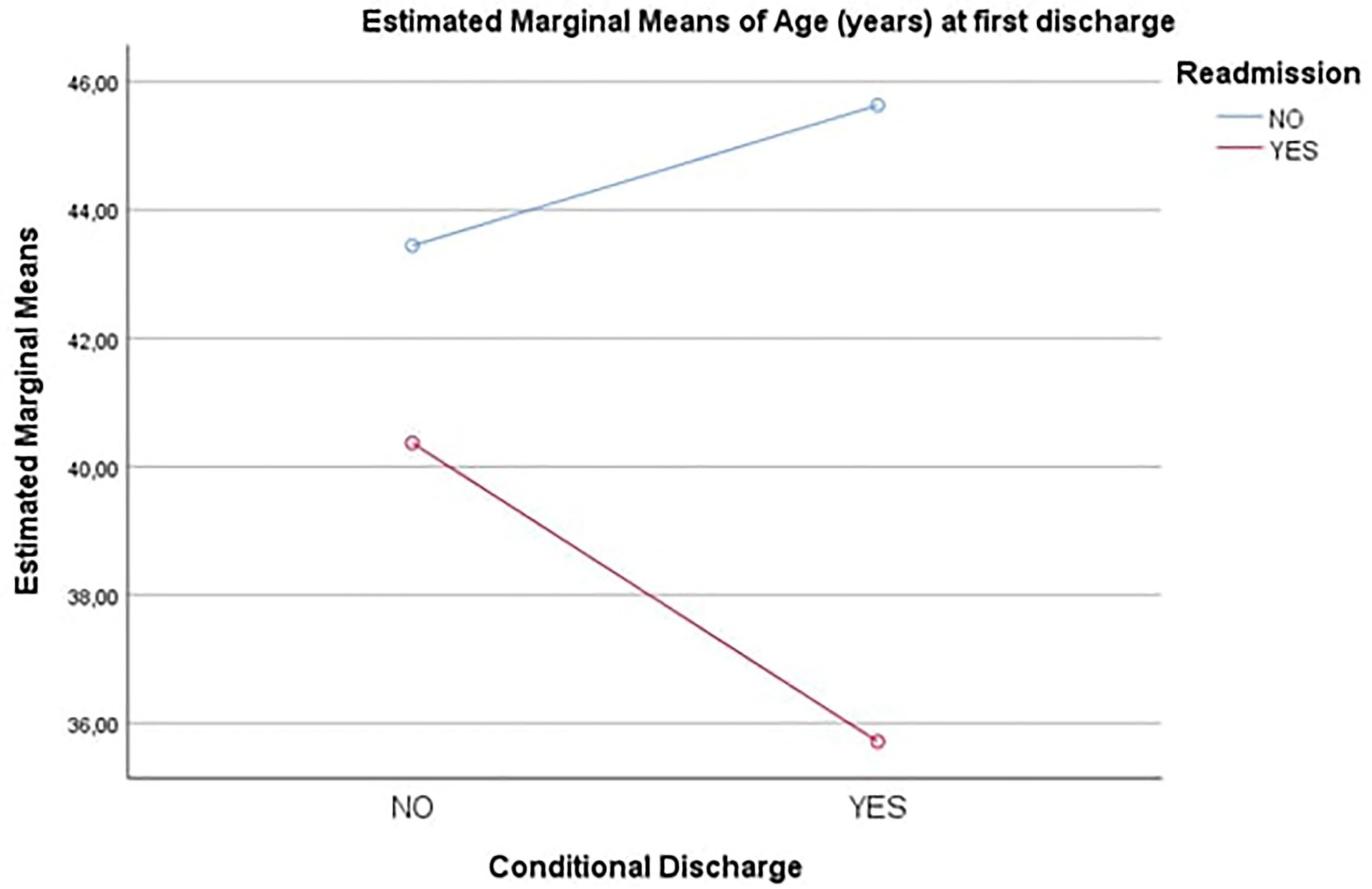
Estimated marginal means – Age (years) at first discharge. Conditional Discharge=Conditional Release.

The differences in the main effects can be observed by evaluating the Estimated Marginal Means (see Figure 4). It can be noted that there were two completely opposite trends: as regards the Readmission group “no,” there was a higher value of Age (years) at first discharge when Conditional Release was “yes” compared to what Conditional Release was “no.” On the contrary as regards the Readmission group “yes,” there was a higher value of Age (years) at first discharge when Conditional Release was “no” than what Conditional Release was “yes.” The fact that the two straight lines are not parallel, however, indicates an interaction effect but, not sufficiently strong, to be statistically significant.

### Crimes Against Person/Against Property

In NRW, 73% of cases committed a crime against a person and 27% a crime against property, while in RW, 50% of cases committed a crime against person and 50% against property (at first admission; see Table 1). Being readmitted or not readmitted depended on whether the crime was against a person or property. Those who committed a crime against a person were readmitted less than the general frequency of readmission (37.5%<46.7%), while those who committed a crime against property were readmitted more often than all who were readmitted (61.8%>46.7%). Cramer’s V was equal to 0.236, so the two variables were only somewhat dependent.

As far the association between Condition (Readmission and Conditional Release) and Crime against the person vs. Crime against property was concerned, the chi-square test turned out to be, albeit slightly, not statistically significant (*p*=0.128). It was not possible therefore to exclude that the difference in percentages of Crime against in the various subgroups may be due to chance.

### Conditional Release Versus Unconditional Release at First Discharge

70.8% of NRW and 46.7% of RW (at the first discharge) were discharged on CR (see Table 1). Being readmitted or not readmitted depended on whether or not the person was discharged on conditional release. Cramer’s V was equal to 0.236, so the two variables were only somewhat dependent.

## Discussion

This is the first study in Italy aimed at comparing two groups of female patients discharged from a FPT: Those who were placed on conditional release and those who were discharged unconditionally.

A key finding of our study was that the readmission into a female REMS depended on the presence of SUD, a primary diagnosis on Axis II, younger age, being unconditionally discharged at the first discharge, having had a shorter length of stay and having committed a crime against property for the first REMS admission but did not depending on a primary diagnosis on Axis II. Also, we found that the median length of treatment in this REMS was shorter in comparison with the international lengths of inpatient treatment.

On Logistic Regression, in facts, by observing the estimated coefficients in Table 5, it can be seen that the statistically significant variables were Substance Use, Length of first stay and Conditional Release at level 0.001 (*p*<0.001), and the variables Crime against and Age (years) at first discharge at level 0.10 (*p*<0.10). Only the Primary Diagnosis at first discharge variable was not statistically significant. Looking at the Odds Ratio (Exp column (b)), the following comments can be made as:

- The use of substances increased the probability of having Readmission Yes by about 23 times compared to Readmission No.
- Having a Crime against “property” compared to “person” increased the probability of having Readmission Yes by 180% compared to Readmission No.
- For each additional month inpatient treatment (Length of stay), the probability of Readmission Yes decreased by 3% compared to Readmission No.
- For each additional year of Age (years) at first discharge, the probability of having Readmission Yes decreased by about 5% compared to Readmission No.
- Being “Conditionally Released” compared to “Unconditionally Released” reduced the probability of having Readmission Yes by 79% compared to Readmission No.

**Table 5:** Logistic regression – variables in the equation.

		B	S.E.	Wald	df	Sig.	Exp(B)
Step 1^a^	Primary diagnosis at first discharge (1)	0.387	0.577	0.451	1	0.502	1.473
	Substance Use (1)	3.161	0.915	11.935	1	0.001	23.604
	Crime against (1)	1.033	0.595	3.015	1	0.082	2.809
	Length of stay (months)	−0.029	0.011	7.475	1	0.006	0.971
	Age (years) at first discharge	−0.050	0.027	3.429	1	0.064	0.951
	Conditional release (1)	−1.543	0.589	6.861	1	0.009	0.214
	Constant	2.372	1.266	3.511	1	0.061	10.718

a *Variable(s) entered on step 1: Primary Diagnosis at first discharge, Substance Use, Crime against, Length of stay (months), Age (years) at first discharge, and Conditional Release*.

Finally, it should be mentioned that the model thereby specified has a much better percentage of correct predictions than the null model. We can therefore state that the variables introduced, with the exception of “Primary Diagnosis at first discharge,” were able to effectively explain the phenomenon of interest (Readmission) and to predict with good results the Readmission category (yes/no).

Therefore, the logistic regression confirmed that there was a statistically significant association between each of the five variables Substance Use Disorders: presence/absence, Length of first stay, Mean age at first discharge, Crimes against person/property and Conditional Release/Unconditional Release at first discharge, and the Readmission/Non-readmission variable, but not between the latter and the Primary diagnosis at first discharge variable.

From further analysis, the intersection between the Conditional Release variable and Readmission variable allowed us to make other observations. For the qualitative variables, Primary Diagnosis at first discharge, Substance Use Disorders, and Crime against person/property, the chi-square test showed that the only variable to be significant for the Readmission was the use of substances in the conditionally released females. This difference, found in women conditionally released and not in those unconditionally released, could be linked to the fact that most women with the presence of SUD were conditionally released and therefore this confirmed that, despite the application of the highest form of legal protection at discharge, the presence of SUD is a major risk factor for readmission to a forensic facility. In regard to the quantitative variables Length of first stay, and Age at first discharge, ANOVA highlighted that for unconditionally released women the factor that mainly affected the readmission was the duration of inpatient treatment, which was significatively longer in non-readmitted compared than to readmitted patients. As for the readmitted women, the difference in length of inpatient treatment did not show significant differences between those conditionally and unconditionally released.

Our similarly conducted study of males, who were conditionally and unconditionally discharged from an Italian REMS (Rossetto et al., unpublished), afforded a unique opportunity for gender comparison. In both males and females, SUD was associated with readmission. In male patients, a diagnosis of personality disorder was associated with readmission and similarly in females, an Axis II diagnosis was associated with readmission. Younger age was positively associated *with readmission* in males and weakly associated in females. In females, consistent with international studies, unconditional discharge and shorter lengths of inpatient treatment were associated with readmission, whereas these parameters were not associated with readmission in our study of readmission of male patients. Crime against property was also weakly associated with the readmission of females, but not males. In the present study, SUD was significantly correlated with readmission of female insanity acquittees released from a security facility. In our companion study of male insanity acquittees, SUD was also associated with readmission.

### Limitations

This study has some important limitations. First, the data were collected retrospectively from an historical cohort. Second, the sample sizes were relatively small, as the subjects came from a comparatively small female REMS population. Moreover, we were unable to assess other clinical factors, such as secondary diagnosis, personality traits, different classes of illegal substance use, social support, adherence with medication, and readmission in psychiatric wards, which are of significant importance to offenders with mental illness (Grann et al., 2008).

Finally, during the study period, we did not obtain clinical and legal information on those who were not readmitted due to the impossibility of accessing clinical and legal databases external to the REMS. Therefore, we were unable to examine the three important outcome measures of mortality, readmission, and violent and non-violent reoffending.

### Interpretation

Some variables should be taken into consideration in the decision-making process that leads to discharge from forensic units, respecting the principle that each patient should be treated at a level of therapeutic security not higher than necessary (Kennedy, 2002). A longer length of treatment in forensic inpatient units and the use of restrictions on discharge are associated with a lower rate of reoffending (Lund et al., 2012; Charette et al., 2015; Jeandarme et al., 2016; Norko et al., 2016), even in female patients (Tully et al., 2019). This is of particular importance in Italy, because the law provides that the duration of the security measure in REMS must be as short as possible and in any case, no longer than the maximum duration of the custodial sentence provided for that offense (excluding offenses for which life imprisonment is a sentencing option) and also considering that the use of risk assessment tools in Italy has been very limited so far.

The number of cases of violently reoffending in females was numerically small, consistent with international literature (Maden et al., 2006), although it was not possible to calculate the rate of reoffending (violent and non-violent).

The finding that crime against property is significantly higher in RW compared to NRW is consistent with literature. With regard to the crime that led to the first admission, the percentage of attempted homicides and homicides was higher among NRW with respect to the RW.

An accurate assessment and risk management should be performed on young women with a diagnosis of personality disorder and substance abuse, because these are the patients who have the greatest risk of being readmitted into a forensic facility. The underestimation of the risk of recidivism and readmission is favored by the non-use in a systematic way of internationally validated instruments for risk assessment and management that constitute an important support for the formation of structured professional judgment. Among these instruments, we can include, for example, the HCR-20 (Douglas et al., 2014) and the DUNDRUM-quartet (O’Dwyer et al., 2011) which seems particularly suitable for the Italian reality. In female patients, without the use of these tools, it can be hypothesized that there is a tendency for clinicians to consider female patients at lower risk of violent recurrence than men. For example, psychopathic females are predominantly diagnosed as having a personality disorder according to DSM-IV-TR (Carabellese et al., 2018).

### Conclusion

Young female patients with personality disorders and substance use require special attention to risk assessment and may need longer treatment in the REMS as well as a well-structured outpatient program including continued substance use rehabilitation and relapse prevention as well as additional specific restrictions when released conditionally. Patients discharged from REMS should be monitored long term in order to measure rates of mortality, readmission, and reoffending. There is evidence in the literature to suggest that some psycho-social factors exert a protective effect. This finding has a direct and immediate impact and requires to be fully considered in order to draw up adequate individual treatment programs; and even more in Italy, where the forensic and general psychiatric public facilities, mainly based on a community model, do not imply long-term internment, but only a short-term one both in forensic treatment and in general psychiatry. Not to be overlooked in future research is the nature and extent of inpatient and outpatient treatment and measures for integrating discharged patients back into the community.

## Data Availability Statement

The original contributions presented in the study are included in the article/supplementary material, further inquiries can be directed to the corresponding author.

## Ethics Statement

The studies involving human participants were reviewed and approved by Poli-REMS Castiglione delle Stiviere, ASST Mantova, Italy. The patients/participants provided their written informed consent to participate in this study.

## Author Contributions

All authors took part in the process of creating this manuscript, satisfying the criteria provided by the editorial standards in relation to authorship. Specifically, IR, FF, and MG gave a more substantial contribute in the writing process, and AF was involved in the conception of the manuscript and gave substantial contributes during the reviewing process, other than being the corresponding author. FC, GV, and FC, were involved in the writing process and in the final approvation of the manuscript. LP gave her contribute in the drafting of the manuscript, specially during the reviewing process. Finally, MC, was dedicated to the analysis and interpretation of data. All authors contributed to the article and approved the submitted version.

## Conflict of Interest

The authors declare that the research was conducted in the absence of any commercial or financial relationships that could be construed as a potential conflict of interest.

## Publisher’s Note

All claims expressed in this article are solely those of the authors and do not necessarily represent those of their affiliated organizations, or those of the publisher, the editors and the reviewers. Any product that may be evaluated in this article, or claim that may be made by its manufacturer, is not guaranteed or endorsed by the publisher.
